# Preparation and Dielectric Properties of SiC/LSR Nanocomposites for Insulation of High Voltage Direct Current Cable Accessories

**DOI:** 10.3390/ma11030403

**Published:** 2018-03-08

**Authors:** Nanqiang Shang, Qingguo Chen, Xinzhe Wei

**Affiliations:** Key Laboratory of Engineering Dielectric and its Application, Ministry of Education, Harbin University of Science and Technology, Harbin 150040, China; qgchen@263.net (Q.C.); weixinzhe1234@163.com (X.W.)

**Keywords:** cable accessories, liquid silicone rubber, SiC/LSR nanocomposites, dielectric properties, electric field distribution, thermal stimulated current test

## Abstract

The conductivity mismatch in the composite insulation of high voltage direct current (HVDC) cable accessories causes electric field distribution distortion and even insulation breakdown. Therefore, a liquid silicone rubber (LSR) filled with SiC nanoparticles is prepared for the insulation of cable accessories. The micro-morphology of the SiC/LSR nanocomposites is observed by scanning electron microscopy, and their trap parameters are characterized using thermal stimulated current (TSC) tests. Moreover, the dielectric properties of SiC/LSR nanocomposites with different SiC concentrations are tested. The results show that the 3 wt % SiC/LSR sample has the best nonlinear conductivity, more than one order of magnitude higher than that of pure LSR with improved temperature and nonlinear conductivity coefficients. The relative permittivity increased 0.2 and dielectric loss factor increased 0.003, while its breakdown strength decreased 5 kV/mm compared to those of pure LSR. Moreover, the TSC results indicate the introduction of SiC nanoparticles reduced the trap level and trap density. Furthermore, the SiC nanoparticles filling significantly increased the sensitivity of LSR to electric field stress and temperature changes, enhancing the conductivity and electric field distribution within the HVDC cable accessories, thus improving the reliability of the HVDC cable accessories.

## 1. Introduction

High voltage direct current (HVDC) transmissions have attracted increasing attention because of their many advantages [1,2,3], such as large capacity, long distance, fast and flexible power regulation, high transient stability, and low line loss. HVDC cables are indispensable to HVDC systems and have been widely used in asynchronous networks, underground power grids, and submarine transmission cables recently [4,5,6].

The operational safety and reliability of HVDC cables is very important for the stability of the HVDC transmission network [7]. Cable accessories have always been the weakest part of HVDC cables because of their complex insulation structure, where most failures occur [8,9]. The composite insulation of cable accessories generally comprises cross-linked polyethylene (XLPE; cable insulation) and silicone rubber (SR; reinforced insulation of accessories). Under DC voltage, the electric field distribution of cable accessories only depends on the conductivity of the composite insulation and the interface space charge [10]. Moreover, the conductivity is severely affected by temperature and electric field strength, while the dependence of two materials is different [11,12,13]. In general, the conductivity of XLPE is one or two orders of magnitude higher than SR insulation, which easily leads to electric field distortion or even insulation breakdown [14,15]. Further, it is difficult to realize uniform electric field distribution of cable accessories by varying the temperature and electric field stress. Nevertheless, the application of nanocomposite dielectrics provides a solution for the conductivity matching problems in HVDC cable accessories.

MgO/XLPE nanocomposites as main insulators for ±500 kV HVDC cables have been successfully developed in Japan [16,17]. ABB Company has added a nonlinear transitional layer between the main insulation of the cable and the reinforced insulation of the cable accessories to match the conductivity of XLPE/SR [18,19]. This additional layer realizes uniform electric field distribution within the cable accessories and reduces the space charge on the interface. Nano modification has also been widely used to improve various properties of materials, such as thermal conductivity, flame retardancy, and dielectric and mechanical properties [20,21,22,23]. Numerous related experimental studies have been reported globally. For example, preparing XLPE composite dielectrics by adding a small amount of nanoparticles (such as SiO_2_, ZnO, Al_2_O_3_, and MgO) has achieved a certain degree of success in improving the insulation properties of these dielectrics [24,25,26] and suppressing the space charge. These nano modification studies mainly focus on cable insulation (XLPE), and research on nano modification of reinforced insulation of cable accessories (SR) has been relatively scarce. In this study, a nanocomposite dielectric was prepared for insulation of cable accessories using SiC nanoparticles as the filler and liquid silicone rubber (LSR) as the matrix, achieve a conductivity matching with XLPE and promoting uniform distribution of the electric field within the DC cable accessories to improve the safety and reliability of HVDC cable accessories.

## 2. Materials and Methods

### 2.1. Sample Preparation

POWERSIL^®^737 manufactured by Wacker Chemical Co., Ltd. (Munich, Germany), was used for sample preparation. This is an A/B two-component addition type LSR and is different from traditionally used high temperature vulcanization or room temperature vulcanization SR because of its low viscosity, excellent mechanical properties, and outstanding dielectric behavior. SiC nanoparticles were obtained from Hefei Kelvin Energy Technology Co., Ltd. (Hefei, China) (purity ≥ 99.9%, average size 60 nm).

**Figure 1:**
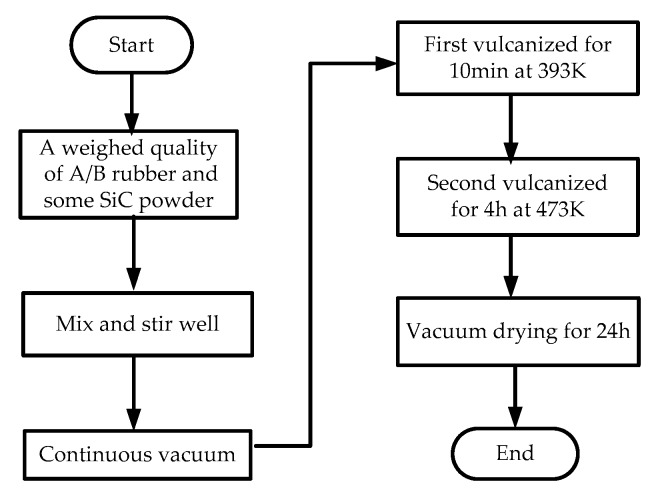
SiC/liquid silicone rubber (LSR) samples preparation.

The flow chart of the preparation of the SiC/LSR nanocomposites is shown in Figure 1. The sample preparation process was as follows: a weighed quantity of A/B rubber and a certain amount of SiC nanoparticles were taken in a beaker and mixed using a multifunctional agitating machine and evacuated repeatedly until no bubbles were generated. Then, the rubber was molded under a flat vulcanizing machine for 10 min at 393 K and 15 MPa; the secondary vulcanization was performed in a circulating air oven with fresh air supply at 473 K for 4 h. Finally, the samples were placed in a drying oven for 24 h to eliminate the impurities and moisture. The samples had a radius of 50 mm and a thickness of 0.3 mm. 

### 2.2. Dielectric Properties

**Figure 2:**
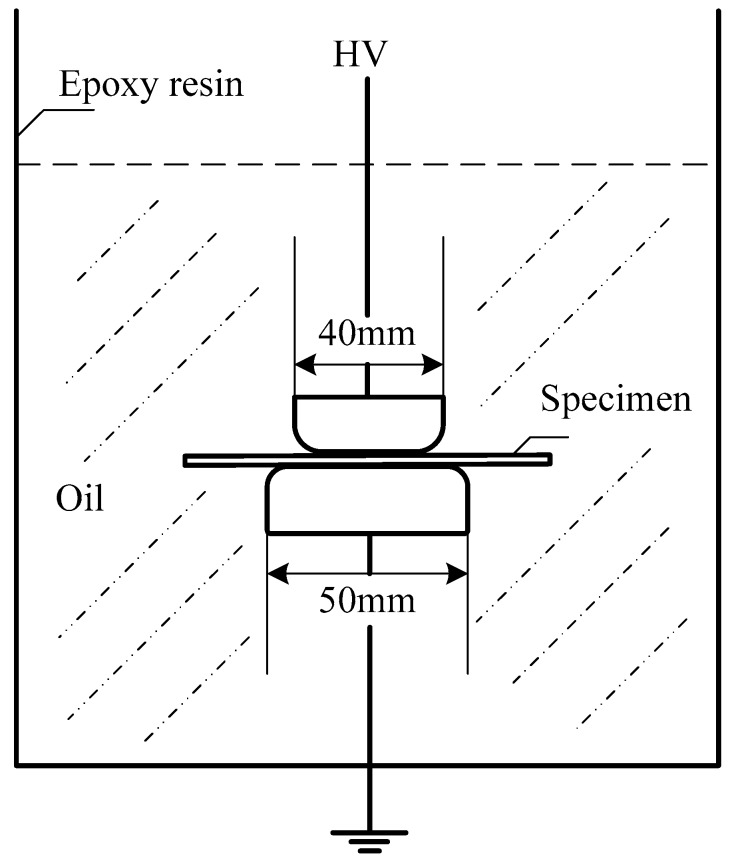
Direct-current (DC) breakdown electric field strength test system.

**Figure 3:**
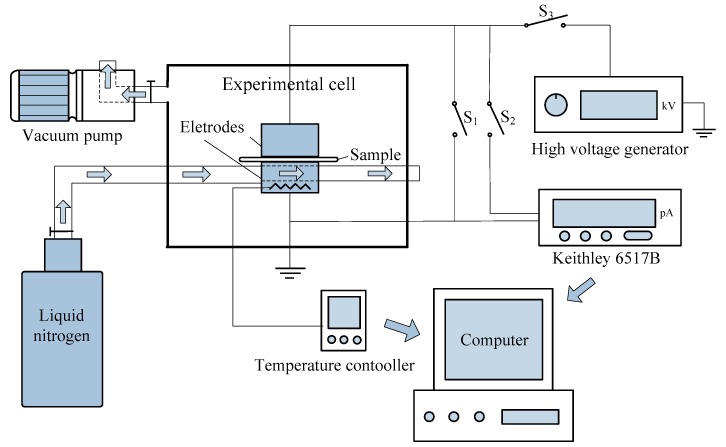
Schematic of thermal simulated current (TSC) measurement system.

**Figure 4:**
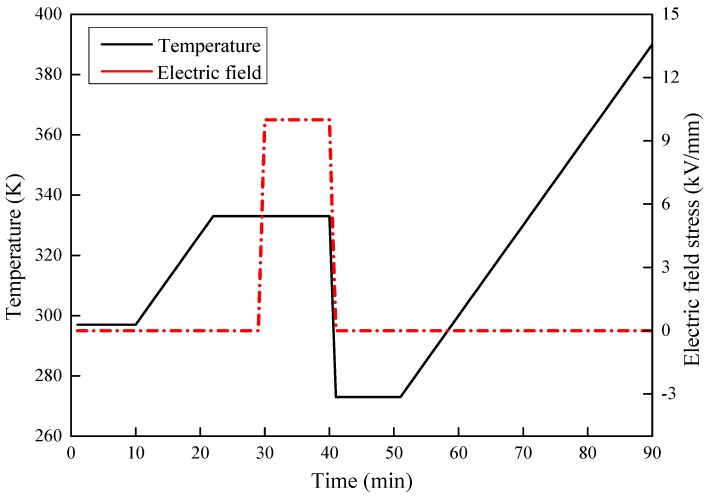
Curves of temperature and electric field stress versus time of TSC test.

The dielectric properties of the pure LSR and SiC/LSR nanocomposites were tested according to the following aspects: 

(a) Conductivity: The DC conductivity of the LSR and SiC/LSR samples was measured at room temperature by a three-electrode system, as shown previously [27]. A high-voltage electrode was connected to a DC source through a series of resistance. The DC source had an output voltage of 0–10 kV to obtain DC electric field conductivity varying from 0.1 to 30 kV/mm. The stable current (I) was recorded after applying the DC voltage for 10 min. For accuracy, multiple samples were employed to ensure repeatability, and the average values were considered.

(b) Dielectric spectrum: The dielectric spectrum of the pure LSR and SiC/LSR samples was tested using a broadband dielectric/impedance spectrometer (Concept 80, Novocontrol Technologies, Montabaur, Germany). The test frequency range was 10^−1^–10^7^ Hz. The samples were deposited on both sides of a gold electrode, and the diameter of the test specimen was 20 mm.

(c) Dielectric strength: The DC breakdown electric field strength of the samples was measured with a two-electrode system, and the entire testing system was placed in an epoxy resin drum with transformer oil (as shown in Figure 2). The test was based on Standardization Administration of China (SAC) Publication No. GB/T 1408.2-2006, which is equivalent to standard IEC 60243. The Weibull distribution was used to characterize the DC breakdown strength after discarding the maximum and minimum values.

(d) Thermal stimulated current (TSC) test: The SiC/LSR samples was polarized under an electric field of 10 kV/mm at 333 K for 10 min to characterize their trap parameters. Afterwards, the temperature was decreased to 273 K quickly using liquid nitrogen until the depolarization current of sample was less than 1 pA. Then, the temperature was linearly increased to 393 K at 3 K/min and the TSC of the sample was measured. The TSC measurement system included a Keithley 6517B electrometer, a DC high voltage generator, vacuum equipment, and heating and cooling systems. The setup of the TSC measurement system and test conditions are shown in Figure 3 and Figure 4, respectively.

## 3. Results and Discussion

### 3.1. Microstructure

**Figure 5:**
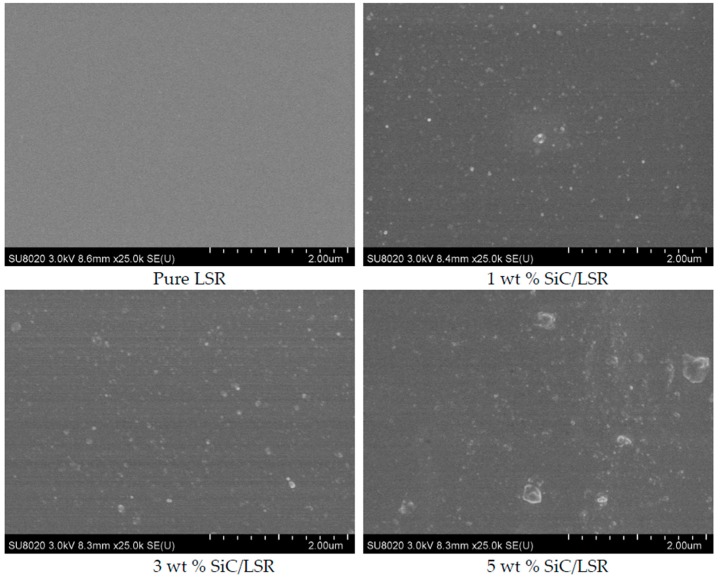
Cross-sectional SEM photographs of pure LSR and SiC/LSR nanocomposites with different SiC concentrations.

The micro morphology of the pure LSR and SiC/LSR nanocomposites with different SiC concentrations was observed using scanning electron microscopy (SEM, SU8020 Hitachi High Technologies Corp., Tokyo, Japan). The central area of observation and the cross-sectional SEM photographs are shown in Figure 5. In the figure, we can observe that the dispersion of the SiC nanoparticles in the matrix decreased with increasing SiC concentration because high concentrations made the nanocomposite prone to agglomeration. 

### 3.2. *Conductivity*

**Figure 6:**
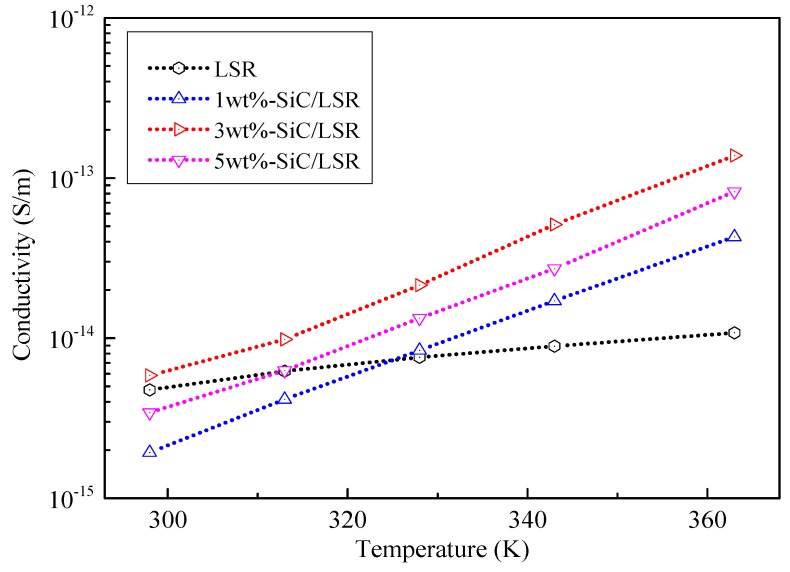
Conductivity vs. temperature of pure LSR and SiC/LSR nanocomposites with different SiC concentrations at 20 kV/mm.

The curve of conductivity vs. temperature of the pure LSR and SiC/LSR nanocomposites with different SiC concentrations at 20 kV/mm is shown in Figure 6. The relationship between the conductivity (*γ*) and temperature of polymers can be expressed by the following equation:

(1) $$\gamma =\gamma_{0}E^{\alpha }$$

where *γ*_0_ is a polymer-related constant and α is the temperature coefficient.

Using Equation (1) for experimental data fitting calculations, the temperature coefficient of pure LSR was 0.12, indicating that the conductivity of pure LSR showed little change with increasing temperature, while the conductivity of XLPE increased by two orders of magnitude with increasing temperature, which would lead to severe electric field distribution distortion and interface space charge accumulation. The temperature coefficient of the SiC/LSR nanocomposites was 0.6. The SEM results showed that agglomeration could occur when the nanoparticles had a higher concentration of SiC; therefore, the 3 wt % SiC/LSR nanocomposites showed highest conductivity and the value increased by more than one order of magnitude with increasing temperature. This indicated that the SiC nanoparticles doping could effectively improve the nonlinear conductivity of LSR by varying the temperature. Thus, the electric field distribution in cable accessories using SiC/LSR nanocomposites is better than that using pure LSR. 

**Figure 7:**
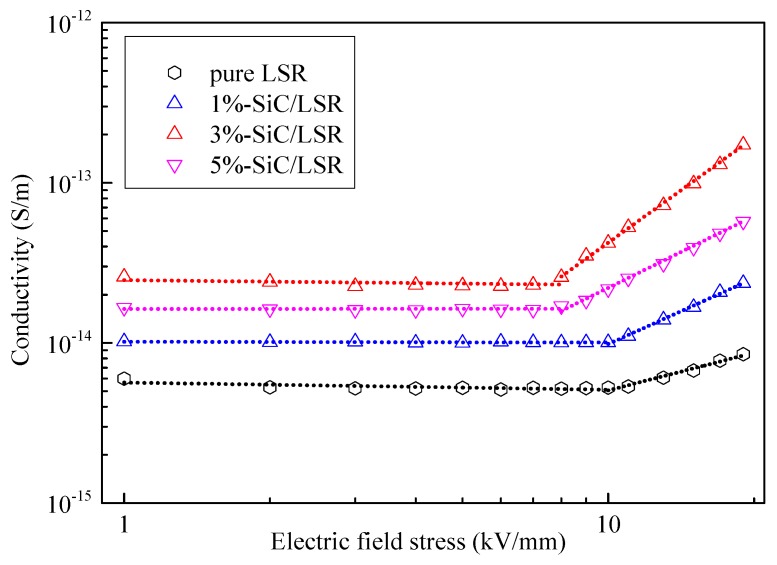
Conductivity vs. electric field stress of pure LSR and SiC/LSR nanocomposites with different SiC concentrations at 70 °C.

The plot of conductivity vs. electric field strength at 70 °C for the pure LSR and SiC/LSR samples with different SiC concentrations is shown in Figure 7. 

The relationship between conductivity and electric field stress can be obtained by the following equation:

(2) $$\gamma =AE^{\beta }$$

By logarithmic transformation of Equation (2), we get

(3) lgγ=lgA+βlgE

where A is a constant related to properties of the material and *β* is the nonlinear coefficient. Thus, there is a linear relationship between lg*γ* and lg*E*, and the slope of the changing curve *β* represents the degree of nonlinear characteristics [27]. Using linear fitting for the two segments, the threshold electric field, the electric filed stress at which nonlinear conductivity is observed, is represented as Ec and the nonlinear coefficient *β* are shown in Table 1.

As shown in Table 1 and Figure 7, the nonlinear coefficient of pure LSR was small and the conductivity showed little change with increasing electric field stress. Further, the nonlinear coefficient of the SiC/LSR samples was several times greater than that of pure LSR, and the value for the sample containing 3 wt % SiC was the highest. As shown in Figure 7, the conductivity of the SiC/LSR samples reached an inflection point at which nonlinear conductivity was observed. The electric field stress value corresponding to this inflection point was called the threshold electric field. The SiC/LSR samples with different SiC concentrations showed different values for the threshold electric field. The 3 wt % SiC/LSR sample exhibited the best nonlinear conductivity and the lowest threshold electric field (about 7 kV/mm). The nonlinear conductivity of pure LSR could be considered insignificant, and its threshold electric field was 12 kV/mm. This indicated that the SiC nanoparticles doping could improve the nonlinear conductivity of LSR and lower its threshold electric field.

The conductivity of insulation material was depended on the concentration of charge carriers, the charge of the charge carriers, and charge mobility. The doping of nanoparticles has a great influence on the charge mobility, while the charge of charge carriers is basically unchanged and the concentration of charger carriers was determined by matrix material. The charge mobility is related to temperature and the jump barrier height needed overcome. In the presence of an external electric field, the jump barrier height could be decreased. When the threshold electric field was reached, the jump barrier height obviously decreased. At this moment, the numbers of carriers increased greatly and rapidly [28,29]. Therefore, a substantial increase in conductivity and polymers showed a nonlinear relationship with the electric field. In addition, the SiC nanoparticles doping led to the overlapping of the interface between adjacent nanoparticles. It is generally considered that the interface has a higher conductivity than the nanoparticles and matrix. In the presence of an external electric field, many charge carriers absorb sufficient energy to cross the potential barrier and participate in the conduction.

The conductivity of the SiC/LSR nanocomposites was higher than that of pure LSR by more than one order of magnitude under the same electric field stress and this difference in conductivity increased with increasing electric field stress. This showed that the addition of the SiC nanoparticles could increase the sensitivity of LSR to electric field stress and reduce the conductivity mismatch between the insulation of the cables and of the cable accessories caused by electric field stress changes, thus improving the electric field distribution within the cable accessories.

### 3.3. Relative Permittivity and Dielectric Loss Factor

**Figure 8:**
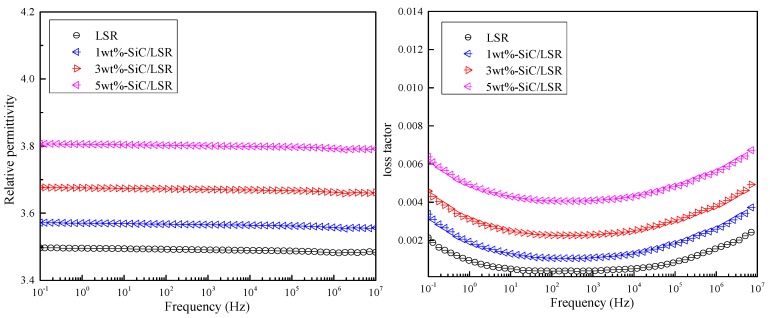
Dielectric spectrum characteristics of pure LSR and SiC/LSR nanocomposites with different SiC concentrations at 25 °C.

The relationship between relative permittivity/dielectric loss factor and frequency of the LSR and SiC/LSR nanocomposites with different SiC concentrations is shown in Figure 8.

In Figure 8, the relative permittivity of the SiC/LSR nanocomposites was higher than that of pure LSR in the given frequency range. Moreover, the SiC/LSR samples with higher SiC concentrations showed a larger relative permittivity. The relative permittivity of the pure LSR and SiC/LSR samples was both a fixed value with the increase of frequency. 

The dielectric loss factor of the pure LSR and SiC/LSR nanocomposites showed the same trend in the given frequency range, and the value of dielectric loss increased with increasing SiC concentration. Both displacement and relaxation polarizations could be established in detail from these results. The dielectric loss factor was high at low frequency (<1 Hz). As the relaxation polarization is too difficult to build, the loss factor decreased and gradually stabilized with increasing frequency (10 Hz < f < 10^4^ Hz). Then, because of the increased displacement polarization, the dielectric loss factor increased at high frequency (10^5^ Hz < f < 10^7^ Hz).

### 3.4. DC Breakdown Strength

**Figure 9:**
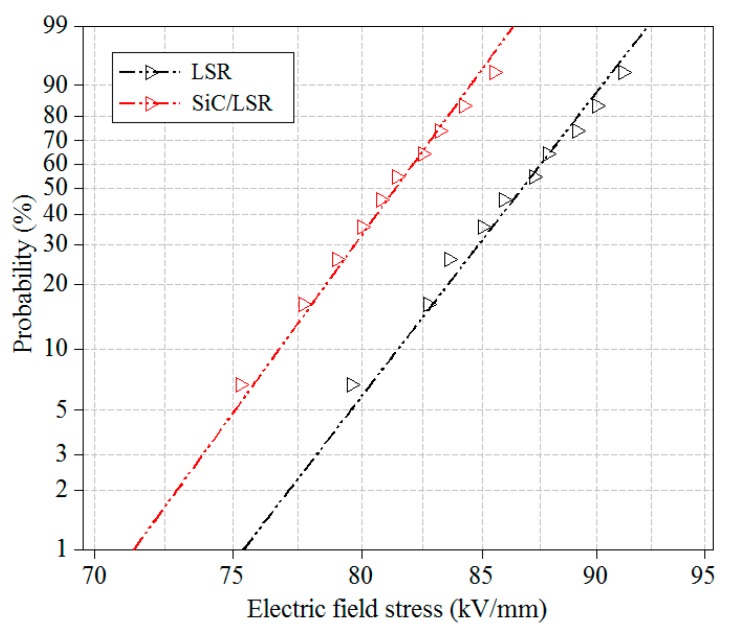
Weibull distribution of DC breakdown strength of pure LSR and 3 wt % SiC/LSR nanocomposites.

The Weibull distribution of the DC breakdown strength of the pure LSR and SiC/LSR samples is shown in Figure 9. It could be seen the breakdown strength of pure LSR was higher than that of the 3 wt% SiC/LSR sample. Because the SiC nanoparticles are semi-conductive, the increased conductivity due to increased carrier mobility led to the formation of an internal discharge path in the presence of an external electric field, which in turn resulted in the decreased breakdown strength of the polymer dielectric.

### 3.5. TSC

**Figure 10:**
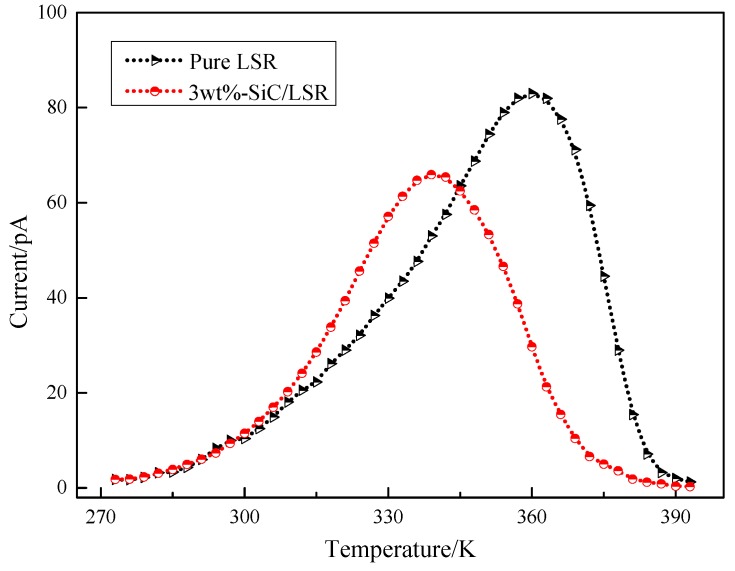
TSC curves of pure LSR and 3 wt % SiC/LSR nanocomposites.

TSC tests were conducted to represent the trap parameters of the LSR nanocomposites. The TSC curves of the pure LSR and 3 wt % SiC/LSR nanocomposites are shown in Figure 10. 

Under high temperature and DC electric field, the migrated carriers of the sample were easily trapped by the polymer. On decreasing the temperature to 273 K rapidly using liquid nitrogen, the trapped carriers were “frozen”. During the subsequent slow warming process, the trapped carriers were able to “escape” because of thermal excitation, and the weak currents were recorded by the 6517B electrometer (Keithley, Cleveland, OH, USA). By analyzing the TSC curve in Figure 10, trapped charge could be obtained by the following equation:

(4) $$Q_{TSC}=\int_{t_{2}}^{t_{1}}I\left(t\right)dt=\frac{60}{\beta }\int_{T_{2}}^{T_{1}}I\left(T\right)\;dT$$

where *I*(*T*) is the TSC current value; *T*_1_ and *T*_2_ are the initial and end temperatures, respectively; and *β* is the temperature increase rate (3 K/min).

Meanwhile, the trap level could be calculated according to the half-width method by the following equation:

(5) $$E=\frac{2.47T_{m}^{2}k}{\Delta T}$$

where *T_m_* is the temperature corresponding to the peak current, ∆*T* is the temperature difference between the two half-peak values, and *k* is the Boltzmann constant [30]. The trap parameters of the pure LSR and SiC/LSR samples are shown in Table 2.

As shown in Table 2 and Figure 9, the trap charge quantity and trap level of the SiC/LSR nanocomposites were lower than those of pure LSR. The trap density and charge trap depth were also decreased due to SiC nanoparticles provide the shallow traps. The trap parameters of the polymer were closely related to their macroscopic dielectric properties [31]. The charge trap depth, which controls the charge mobility, represents the required energy for carriers to jump from the trap energy level to the specific energy level in which they can participate in electric conduction in nanocomposite. Therefore, the probability of the charge carriers trapping of the SiC/LSR nanocomposites was reduced, making more carriers participate in the conduction easily, thereby increasing the charge mobility. Thus, the SiC/LSR nanocomposites have better nonlinear conductivity and the reduction in energy required corresponds to the lower threshold electric field of SiC/LSR nanocomposites. Specifically, the DC conductivity was inversely proportional to the trap charge and the breakdown strength was directly proportional to the trap level. Thus, the results of this study are in complete agreement with previous results [32]. In summary, SiC nanoparticles doping could improve the sensitive dependence of LSR to electric field stress and temperature changes while lowering the trap level and decreasing the number of carriers trapped. As a result, the number of charge carriers involved in electric conduction substantially increased microscopically and the conductivity of SiC/LSR nanocomposites significantly increased macroscopically. This was beneficial for current matching with XLPE cable insulation and uniformly improved the electric field distribution in cable accessories, thus improving their safety and reliability.

## 4. Conclusions

Based on the experimental study of the dielectric properties of SiC/LSR nanocomposites, the following conclusions can be drawn:

(1) SiC nanoparticles doping decreases the breakdown strength of LSR, greatly increases its conductivity, and increases its relative permittivity and dielectric loss factor.
(2) SiC/LSR nanocomposites have better nonlinear conductivity characteristics than pure LSR, as their temperature coefficient and nonlinear coefficients are greatly improved, which in turn makes the distribution of the electric field more uniform in HVDC cable accessories.
(3) The introduction of SiC nanoparticles leads to the decrease in the trap level and trap charge of LSR, which affects the transport of carriers in the polymers. The TSC test verifies that the trap parameters have an important effect on the macroscopic dielectric properties of the polymer.

## Figures and Tables

**Table 1:** Nonlinear parameters of pure LSR and SiC/LSR nanocomposite samples.

Sample	Ec (kV/mm)	*β*
LSR	12	0.32
1 wt % SiC/LSR	10	0.58
3 wt % SiC/LSR	7	1.63
5 wt % SiC/LSR	8	1.21

**Table 2:** Trap parameters of pure LSR and 3 wt % SiC/LSR samples.

Sample	Peak Value Current (pA)	Peak Value Temperature (K)	Trap Charge Quantity (nC)	Trap Level (eV)
LSR	82.9	360	80.78	0.614
3 wt % SiC/LSR	65.9	339	59.80	0.583
